# Genome Sequences of Foot-and-Mouth Disease Virus O/ME-SA/Ind-2001e Strains Isolated in Pakistan

**DOI:** 10.1128/MRA.00165-20

**Published:** 2020-04-30

**Authors:** Hayley M. Hicks, Jemma Wadsworth, Mehreen Azhar, Muhammad Afzal, Shumaila Manzoor, Muhammad Abubakar, Ehtisham-ul-Haq Khan, Donald P. King, Nick J. Knowles

**Affiliations:** a The Pirbright Institute, Surrey, United Kingdom; b Food and Agriculture Organization of the United Nations, Pakistan Office, Islamabad, Pakistan; c National Veterinary Laboratories, Islamabad, Pakistan; d Livestock and Dairy Development Department, Government of Punjab, Rawalpindi, Pakistan; KU Leuven

## Abstract

The genome sequences of two foot-and-mouth disease type O viruses isolated from outbreaks of disease in cattle in Pakistan in 2019 are described. They were identified as belonging to serotype O, Middle East-South Asia topotype, Ind-2001 lineage, and e sublineage and represent the first identification of this lineage in Pakistan.

## ANNOUNCEMENT

Foot-and-mouth disease virus (FMDV) causes a highly contagious vesicular disease of cloven-hooved animals leading to livestock production losses and disruption to international trade. Due to a high mutation rate, new FMDV lineages constantly emerge and pose challenges to control strategies. Here, we report the genome sequences of two FMDV isolates generated from vesicular epithelium collected from cattle in Pakistan (Punjab province) using primary bovine thyroid cells (1) and identified as serotype O using an enzyme-linked immunosorbent assay (ELISA) (2). Total RNA was extracted from cell culture supernatants using an RNeasy minikit (Qiagen) (3), and first-strand cDNA synthesis (reverse transcription) was performed using the Superscript III first-strand synthesis system (Life Technologies) (3). Second-strand synthesis was undertaken using 20 μl of cDNA with a second-strand synthesis kit (New England Biolabs [NEB]) (3). One nanogram of each double-stranded DNA (dsDNA) sample was used to prepare sequencing libraries using the Nextera XT DNA sample preparation kit (Illumina) (3). All kits were used according to the manufacturer’s instructions. Sequencing libraries were run on a MiSeq system (Illumina) as previously described (3). A paired-end sequencing run of 2 × 150-nucleotide (nt) read lengths generated 1,342,316 (strain O/PAK/1/2019) and 1,148,186 (strain O/PAK/2/2019) reads. Sequences were mapped against the genome sequence of strain O/BHU/9/2016 (GenBank accession no. MG983691) using SeqMan NGen software with default quality trimming settings and were visualized using SeqMan Pro (Lasergene package version 16; DNAStar, Inc.). Mapping resulted in 56,173 and 39,415 reads making up the genomes of O/PAK/1/2019 (8,187 nt; G+C content, 54.0%; median coverage, 2,518×) and O/PAK/2/2019 (8,187 nt; G+C content, 54.0%; median coverage, 1,393×), respectively. Seven nucleotides at the 5′ end of each genome were not determined, but a short region of the 3′ poly(A) tail was sequenced. An artificial poly(C) tract consisting of 10 Cs was inserted at position 362 [FMDV has a long poly(C) tract of variable length located at this position in the 5′ untranslated region (UTR)]. A single, large open reading frame of 6,999 nt was predicted to encode a polyprotein of 2,333 amino acids containing 4 structural and 10 nonstructural proteins.

**FIG 1:**
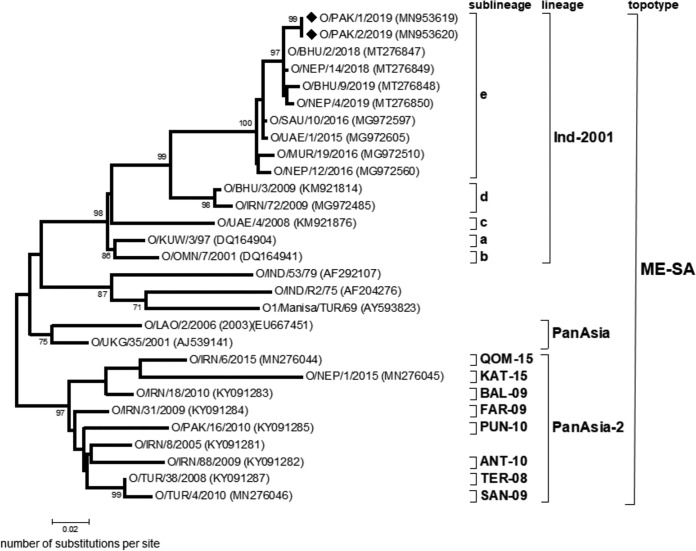
Midpoint-rooted maximum likelihood tree of the VP1 coding region. The tree was produced using MEGA7 (9), and the evolutionary history was inferred based on the Hasegawa-Kishino-Yano model with a discrete gamma distribution to model evolutionary rate differences among sites (5 categories [+G, parameter = 0.3498]). The percentage of trees in which the associated taxa clustered together (by bootstrap analysis) is shown next to the branches (only values of 70% and above are shown). The tree shows representative reference viruses representing lineages and sublineages within the Middle East-South Asia (ME-SA) topotype. The two Pakistan sequences are indicated by black diamonds.

Phylogenetic analysis of the VP1 coding sequence showed that the two viruses belonged to the O/ME-SA/Ind-2001e lineage and revealed a close relationship (>99% nucleotide identity) to recent type O viruses from Bhutan and Nepal (Fig. 1) and India (information provided by the Indian Council of Agricultural Research-Directorate of FMD). The O/ME-SA/Ind-2001 lineage is classified into five sublineages named a, b, c, d, and e, each of which has circulated in South Asia, replacing the previously dominant O/ME-SA/PanAsia lineage (4). In 2013 to 2015, the O/ME-SA/Ind-2001d sublineage emerged to cause outbreaks across North Africa, the Arabian Gulf states, and Southeast Asia (5–7). These events were mirrored in 2015 to 2017 by new introductions of the O/ME-SA/Ind-2001e sublineage into the Arabian Gulf states, Southeast and East Asia, and the normally FMD-free islands of Mauritius (8).

The O/ME-SA/Ind-2001 lineage has not previously been identified in Pakistan or in neighboring countries to the west, i.e., Afghanistan and Iran, except for a single report of O/ME-SA/Ind-2001d in Iran in 2009 (8). Since that time, a total of 669 type O virus isolates from these three countries have been characterized at the World Reference Laboratory for FMD (WRLFMD; Pirbright, UK) without any Ind-2001 viruses being identified. These new sequences are important, since it is possible that this virus could rapidly spread through the region, increasing the complexity of the FMD control situation. These findings and the recent appearance and spread of the O/ME-SA/Ind-2001e lineage elsewhere in Asia (8) reinforce the need to monitor the emergence of new viruses in order to develop appropriate diagnostic and vaccination strategies.

### Data availability.

The nucleotide sequences of FMDV O/PAK/1/2019 and O/PAK/2/2019 have been deposited in GenBank under the accession no. MN953619 and MN953620, respectively. The raw sequence data were deposited in the NCBI Sequence Read Archive under BioProject PRJNA601307.
